# Impact of waiting time for treatment on the progression of superficial esophageal squamous cell carcinoma: a single-center retrospective study

**DOI:** 10.1007/s10388-025-01156-1

**Published:** 2025-09-17

**Authors:** Hidehito Maeda, Fumisato Sasaki, Naohiro Koyoshi, Shohei Uehara, Takahiro Sakae, Akihito Tanaka, Makoto Hinokuchi, Shiho Arima, Shinichi Hashimoto, Shuji Kanmura

**Affiliations:** Digestive and Lifestyle Diseases, Kagoshima University Graduate School of Medical and Dental Sciences, Kagoshima, Japan

**Keywords:** Esopageal cancer, ESD, Wating time

## Abstract

**Background:**

We aimed to identify an acceptable waiting period between the diagnosis of superficial esophageal squamous cell carcinoma (SESCC) and endoscopic submucosal dissection (ESD).

**Methods:**

This retrospective, single-center study included 423 patients with 514 SESCC lesions. All patients underwent image-enhanced magnifying endoscopy at initial evaluation and on the day of ESD. Following three outcomes were assessed: the diagnostic accuracy of tumor invasion depth on the day of ESD using the Japanese Esophageal Society classification; changes in diagnosis between initial and final evaluations across different waiting intervals; and 5-year survival rates based on intrapapillary capillary loop patterns (B1 vs. B2) and waiting duration.

**Results:**

The diagnostic accuracy was 96.6% for B1 vessels (EP/LPM), 59.6% for B2 vessels (MM/SM1), and 84.6% for B3 vessels (SM2 or deeper). Among B1 lesions, 100% remained B1 on the day of ESD across all time groups. For B2 lesions, stability was observed in 100% of cases within one month, 98.2% in one to two months, 92.9% in two to three months, and 100% after three months. Five-year survival rates showed no significant differences among waiting period groups in both B1 and B2 categories.

**Conclusions:**

A waiting period of 3 months is acceptable for patients with SESCC classified as B1 vessels, provided the diagnostic accuracy is maintained. Thorough endoscopic evaluation supports safe scheduling flexibility without adversely affecting long-term outcomes.

**Supplementary Information:**

The online version contains supplementary material available at 10.1007/s10388-025-01156-1.

## Introduction

Endoscopic submucosal dissection (ESD) is the first-line treatment for superficial neoplasms of the esophagus, stomach, and colorectum. Its indications have recently expanded to include lesions in the pharynx and duodenum [1–3], leading to a steady increase in annual ESD procedures. However, constraints in medical resources usually result in unavoidable treatment delays.

The acceptable waiting periods for early gastric cancer have been largely explored [4, 5]; however, limited evidence exists for esophageal cancer, which is believed to progress more rapidly than do neoplasms in other gastrointestinal organs [6]. Tumor progression during the waiting period may preclude curative ESD, necessitating more invasive treatments such as surgery or radiotherapy [7].

In addition, delayed treatment may cause significant psychological distress for patients and healthcare providers. Therefore, understanding the impact of waiting time on tumor progression in superficial esophageal squamous cell carcinoma (SESCC) is essential to optimize scheduling and resource allocation. In this study, we aimed to evaluate whether tumor invasion depth changes during the waiting period before ESD in patients with SESCC.

## Materials and methods

### Patients

This single-center, retrospective study included patients who underwent ESD for SESCC between January 2009 and December 2023. A total of 423 patients with 514 lesions were included. In all cases, the intrapapillary capillary loop (IPCL) patterns were assessed through image-enhanced magnifying endoscopy (IEE-ME) at initial diagnosis and on the day of ESD. Patients who received radiotherapy or chemotherapy during this period were excluded. The study adhered to the Declaration of Helsinki and was approved by the Institutional Review Board of Kagoshima University (Approval No. 180332). Informed consent was obtained from all patients per the institutional guidelines.

### Japan esophagus society magnifying endoscopic classification

Microvascular irregularities were assessed via combined IEE-ME and narrow-band imaging or blue laser imaging. Examinations were performed using upper gastrointestinal endoscopes (GIF-H240Z, H260Z, XZ1200, EZ1500; Olympus, Tokyo, Japan, and EG-L600ZW7; Fujifilm, Tokyo, Japan) and video systems (EVIS LUCERA CV-260SL, EVIS LUCERA ELITE, EVIS X1; Olympus, and LASEREO 7000; Fujifilm). Tumor invasion depth was classified according to the magnifying endoscopic system of the Japanese Esophagus Society (JES) [8].

**Fig. 1:**
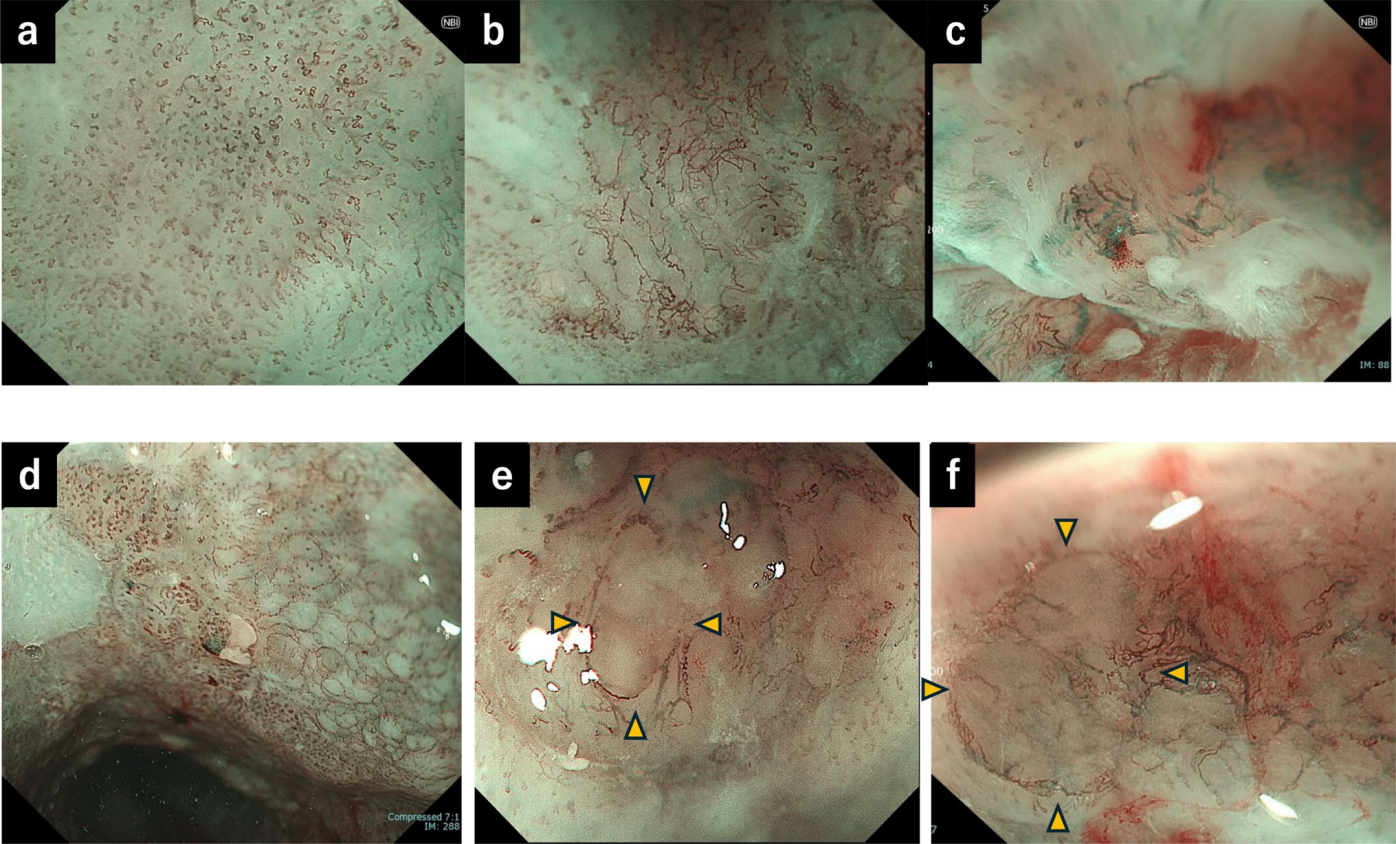
Classification of type B micro vessels and corresponding invasion depths. **a** B1 vessels: EP/LPM invasion. **b** B2 vessels: MM/SM1 invasion. **c** B3 vessels: SM2 invasion. **d** Avascular area (AVA)-small: EP/LPM invasion. **e** AVA-middle: MM/SM1 invasion. **f** AVA-large: SM2 invasion

Type B vessels, defined as abnormal microvessels with marked irregularity or dilation, were subclassified as B1, B2, or B3 (Fig. 1). B1 vessels indicate invasion limited to the epithelium or lamina propria mucosa (T1a-EP or T1a-LPM) (Fig. 1a). B2 vessels correspond to the invasion of the muscularis mucosa or superficial submucosa ≤ 200 μm (T1a-MM or T1b-SM1) (Fig. 1b). B3 vessels indicate submucosal invasion > 200 μm (T1b-SM2) (Fig. 1c). In addition, avascular areas (AVAs) were evaluated. AVA-small surrounded by B1 vessels were classified as B1 type and considered EP/LPM (Fig. 1d). AVA-middle and AVA-large surrounded by B2 vessels were classified as B2 type and considered MM/SM1 (Fig. 1e). AVAs surrounded by B3 vessels were classified as B3 type and considered SM2 (Fig. 1f), based on previous classification [8]. All available magnifying endoscopy images for each lesion were blindly evaluated through a consensus discussion by three board-certified endoscopists, each with over 10 years of experience. Tumor location was determined according to the Japanese Classification of Esophageal Cancer and categorized as the esophageal orifice, cervical esophagus, upper thoracic esophagus, middle thoracic esophagus, lower thoracic esophagus, or the esophagogastric junction zone [9].

### Endpoint

The primary endpoint was the rate of change in invasion depth classification using the JES system, comparing preoperative and day-of-ESD evaluations across different waiting periods. Additionally, diagnostic accuracy on the day of ESD was assessed by comparing endoscopic and histopathologic findings.

The secondary endpoint was the 5-year survival rate stratified by waiting period and preoperative IPCL classification (B1 vs. B2).

### Statistical analysis

Pathological consistency of preoperative IPCL classification across different waiting periods was evaluated using the chi-square test. For the comparison of characteristics by waiting period in B1- and B2-classified SESCC cases, the chi-square test was used for categorical variables, and the Kruskal–Wallis test was performed for continuous variables after assessing normality using the Shapiro–Wilk test. Furthermore, 5-year survival rates were estimated using the Kaplan–Meier method. Group comparisons were conducted using the log-rank test. A p-value < 0.05 was considered statistically significant. Analyses were performed using SPSS software, version 22 (IBM Corp., Armonk, NY, USA).

## Results

### Patient and lesion characteristics

The median patient age was 71 years; 91.3% and 8.7% of patients were male and female, respectively. The median daily alcohol intake was 54 g, and 83.0% of the participants had a history of smoking. Lesion locations included 1.0 and 0.2% at the esophageal orifice and esophagogastric junction, respectively and 3.1, 20.1, 55.0, and 20.9% in the cervical esophagus, upper thoracic esophagus, middle thoracic esophagus, lower thoracic esophagus, respectively. Regarding circumferential orientation, 21.0, 21.8, 28.8, and 26.5% were on the anterior wall, right wall, posterior wall, and left wall, respectively, and 1.9% involved the entire circumference. The lesion involvement of the luminal circumference was as follows: 0–1/4 in 41.2%, 1/4–1/2 in 42.4%, 1/2–3/4 in 11.1%, 3/4–1 in 3.3%, and full circumference in 1.9%. The median lesion length was 20 mm. Macroscopic types were 0–I in 0.2%, 0–IIa in 8.2%, 0–IIb in 40.3%, and 0–IIc in 51.4%. Histopathological invasion depth was EP in 33.9%, LPM in 45.5%, MM in 13.0%, SM1 in 3.1%, and SM2 in 4.5% (Table 1).

**Table 1:** Patient and lesion characteristics

Age, median (years)	71 (40–89)
Sex (M/F), n	386/37
Daily alcohol consumption (g), median	54 (0–412)
Smoking (yes/no), n	351/72
Location of lesions, n O/Ce/Ut/Mt/Lt/Jz Ant/Right/Post/Left/Circ	5/16/103/282/107/1 108/112/148/136/10
Circumferential extent of the lesions, n 0–1/4, 1/4–1/2, 1/2–3/4, 3/4–1, 1	212/218/57/17/10
Tumor size (mm), median	20 (2–85)
Macroscopic type, n 0–Ⅰ/0–Ⅱa/0–Ⅱb/0–Ⅱc	1/42/207/264
Depth of invasion, n EP/LPM/MM/SM1/SM2	174/234/67/16/23

*O* esophageal orifuce, *Ce* cervical esophagus, *Ut* upper thoracic esophagus, *Mt* middle thoracic esophagus, *Lt* lower thoracic esophagus, *Jz* zone of the esophagogastric junction, *Ant* anterior, *Post* posterior, *Circ* circumference, *EP* carcinoma in situ, *LPM* lamina propria mucosa, *MM* muscularis mucosa, *SM1* submucosa to a depth of ≤ 200 μm from the muscularis mucosa, *SM2* submucosa to a depth > 200 μm

To assess the potential for selection bias in ESD scheduling, we compared clinical characteristics—including age, sex, lesion location, circumferential extent of lesions, tumor size, macroscopic type, and sedation method—across the different waiting periods. Most variables exhibited no statistically significant differences among the groups (Supplemental Tables 1 and 2). However, among patients preoperatively diagnosed with B1 vessels, a significant difference in sedation method was observed between the < 1 and 1–2 month groups (p < 0.001).

### Diagnostic accuracy of invasion depth using the JES classification

On the day of ESD, the diagnostic accuracy of the JES classification was 96.6% for B1 vessels (EP/LPM), 59.6% for B2 vessels (MM/SM1), and 84.6% for B3 vessels (SM2 or deeper).

### Changes in diagnostic classification by waiting period

Lesions initially classified as B1 vessels remained B1 at ESD in 100% of cases across all intervals: within 1 month (95.4–100%), 1–2 months (97.7–100%), 2–3 months (93.9–100%), and > 3 months (77.3–100%).

For B2 lesions, proportions remaining B2 at ESD were 100% (90.2–100%) within 1 month, 98.2% (89.5–100%) for 1–2 months, 92.9% (66.5–100%) for 2–3 months, and 100% (45.4–100%) for > 3 months. Two lesions initially identified as B2 were reclassified as B1 at ESD.

To evaluate the histopathological consistency of preoperative endoscopic diagnosis across different waiting intervals, we compared the preoperative IPCL classification (B1 or B2) with the final pathological diagnosis. Among lesions classified as B1, the proportion ultimately diagnosed as EP or LPM remained comparable to the diagnostic accuracy presented in Table 2 across all waiting periods, with no significant differences observed. Similar findings were obtained for lesions classified as B2 (Table 4). In addition, the supplemental analysis (Supplemental Table 3) for comparing preoperative IPCL classification with histological depth of invasion at the time of ESD demonstrated diagnostic performance comparable to that presented in Table 2.

**Table 2:** Diagnostic accuracy of invasion depth using the JES classification

Magnifying endoscopic diagnosis	pEP/LPM	pMM/SM1	pSM2
cEP/LPM (B1 vessels)	96.6% (374/387)	3.4% (13/387)	0% (0/387)
cMM/SM1 (B2 vessels)	29.8% (34/114)	59.6% (68/114)	10.5% (12/114)
cSM2 (B3 vessels)	0% (0/13)	15.4% (2/13)	84.6% (11/13)

*EP* carcinoma in situ, *LPM* lamina propria mucosa, *MM* muscularis mucosa, *SM1* submucosa to a depth of ≤ 200 μm from the muscularis mucosa, *SM2* submucosa to a depth > 200 μm

### 5-Year survival rates by waiting period

**Fig. 2:**
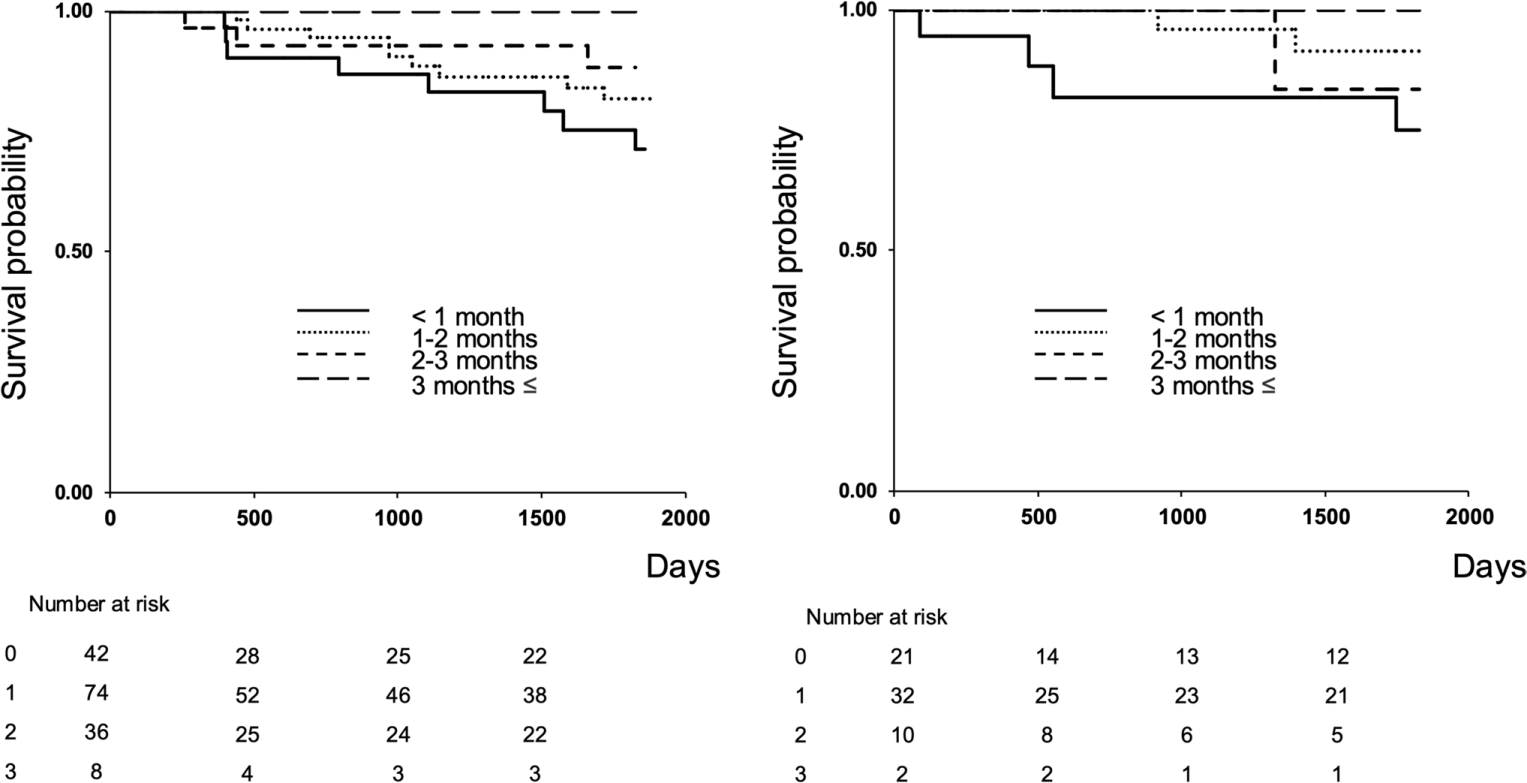
Five-year survival rates by waiting period. **a** No significant differences in 5-year survival across waiting periods in patients with a preoperative diagnosis of B1 vessels (p = 0.37); **b** No significant differences in 5-year survival across waiting periods in patients with a preoperative diagnosis of B2 vessels (p = 0.46)

In the B1 and B2 groups, there were 20 and 7 events (deaths), the median follow-up durations were 1659 and 1807 days, and the censoring rates were 87.5% and 89.2%, respectively. No significant differences were found in the 5-year survival rates across waiting periods for patients diagnosed preoperatively with B1 (p = 0.37) or B2 (p = 0.46) vessels (Fig. 2) (Tables 3, 4).

**Table 3:** Changes in diagnostic classification by waiting period

	< 1 month	1–2 months	2–3 months	> 3 months
B1 vessels preoperative → B1 vessels at the time of ESD (%, n) [95% CI]	100% (97 of 97) [95.4–100]	100% (200 of 200) [97.7–100]	100% (72 of 72) [93.9–100]	100% (16 of 16) (77.3–100)
B2 vessels preoperative → B2 vessels at the time of ESD (%, n) [95% CI]	100% (43 of 43) [90.2–100]	98.2% (54 of 55) [89.5–100]	92.9% (13 of 14) [66.5–100]	100% (4 of 4) [45.4–100]

*CI* confidence interval

**Table 4:** Pathological consistency of preoperative IPCL classification across waiting periods

	< 1 month	1–2 months	2–3 months	> 3 months	p
B1 vessels preoperative → pEP/LPM (%, n) [95% CI]	97.9% (95 of 97) [92.3–99.9]	96.0% (192 of 200) [92.2–98.1]	97.2% (70 of 72) [89.8–99.8]	93.8% (15 of 16) [69.7–100]	0.74
B2 vessels preoperative → pMM/SM1 [95% CI]	68.2% (30 of 43) [53.4–80.1]	52.7% (29 of 55) [39.8–65.3]	50.0% (7 of 14) (26.8–73.2)	100% (2 of 2) (15.0–85.0)	0.23

*EP* carcinoma in situ, *LPM* lamina propria mucosa, *MM* muscularis mucosa, *SM1* submucosa to a depth of ≤ 200 μm from the muscularis mucosa

## Discussion

ESD, developed in Japan in the 1990 s, is a common minimally invasive treatment for early gastrointestinal cancers [10]. Approximately 90,000 ESD procedures are performed annually in Japan, according to the 8th National Database Open Data Report [11]. At our institution, ESD cases exceed 250 annually, with esophageal procedures comprising approximately 35%. Many patients with esophageal lesions have a history of heavy alcohol consumption, making conscious sedation impractical [12, 13]. For lesions ≥ 20 mm or in patients who cannot remain still, general anesthesia is used. The method reduces intraoperative complications, such as perforation and aspiration pneumonia, by limiting patient movement [14–17], and is generally accepted by patients and clinicians. However, this approach extends waiting periods owing to limited operating room availability.

Our findings indicate that for lesions with a pre-ESD B1 classification, diagnostic consistency was preserved even with waiting periods of 3 months. Furthermore, across all waiting intervals, the diagnostic accuracy remained comparable to that reported in Table 2. This supports the safety of scheduling ESD within this timeframe without compromising accuracy or outcomes. Moreover, providing such evidence may alleviate anxiety among patients concerned about delays.

In this study, the JES classification was used to assess invasion depth as follows: B1, B2, and B3 vessels corresponded to EP/LPM, MM/SM1, and SM2 or deeper, respectively. Previous reports cited diagnostic accuracies of 92.4% (B1), 55.7% (B2), and 90.7% (B3) [7], comparable with our results indicating 96.6%, 59.6%, and 84.6%, respectively. Notably, B1 classifications remained stable in over 90% of cases despite extended waiting periods, supporting the clinical safety of delayed ESD for superficial lesions.

In addition, B2 lesions showed minimal diagnostic change across intervals. However, the lower accuracy, smaller sample sizes, and the classification of pMM and pSM1 as B2 lesions—each requiring distinctly different management strategies—preclude definitive conclusions. Further studies involving more B2 cases are warranted for a robust evaluation. Additionally, in two lesions initially diagnosed as B2 and subsequently classified as B1 at the time of ESD, the IPCLs observed during the initial evaluation possibly corresponded to the B2i subtype [18]. This potential ambiguity highlights the inherent challenges in IPCL classification and suggests the possibility of minor discrepancies in interpretation, even among experienced endoscopists. Lesions classified as B3 were excluded, as SM2 invasion typically contraindicates ESD.

Furthermore, 5-year survival analysis revealed no significant variation across waiting periods for patients diagnosed preoperatively with B1 or B2 vessels, suggesting that moderate treatment delays do not adversely affect long-term outcomes.

However, this study has some limitations. First, this was a retrospective, single-center study. Second, cases with waiting periods of > 3 months were limited. Because patients with SESCC usually have synchronous head and neck cancers [19], future data on longer delays may clarify how to prioritize interventions across multiple sites. Third, we evaluated only invasion depth and did not assess changes in lesion size in the present study. While lesion size does not predict lymph node metastasis, increased circumferential extension complicates procedures and exacerbates the risk of post-ESD strictures [7]. Lesions ≥ 5 cm in circumferential length are particularly associated with strictures, possibly requiring alternative treatment strategies. Future studies should include lesion size as a variable. Fourth, owing to the retrospective design of this study, variations in the quality and quantity of magnifying endoscopy images between the initial evaluation and the day of ESD cannot be completely excluded. In particular, procedural constraints during ESD may have affected image acquisition. To minimize potential bias, we included only cases where magnifying endoscopic findings could be clearly and reliably evaluated at both time points. Fifth, although this study was retrospective and subject to potential selection bias, we compared baseline clinical characteristics across waiting time groups to address this concern. Among patients preoperatively diagnosed with B1 vessels, a statistically significant difference was observed in sedation methods between the < 1 and 1–2 month groups. Notably, only two cases demonstrated a change in vessel classification during the waiting period, making statistical adjustment unfeasible. Finally, rare pathological subtypes such as basaloid squamous, adenosquamous, and neuroendocrine carcinomas were not evaluated. These are more aggressive than typical squamous cell carcinoma and require earlier intervention [20–22]. Although they usually exhibit reticular (Type R) vascular patterns on IPCL classification [8, 23], B1-type vessels may also appear in surrounding tissues, necessitating caution [20–22].

## Conclusions

For superficial SESCCs diagnosed preoperatively with B1 vessels, a waiting period of ≤ 3 months appears acceptable, as no progression in invasion depth was observed. These findings highlight the value of efficient treatment planning that accommodates ESD demand within the constraints of limited medical resources.

## Supplementary Information

Below is the link to the electronic supplementary material.

Supplementary file1 (DOCX 21 KB)

## Data Availability

The datasets generated and/or analyzed during the current study are available from the corresponding author on reasonable request.
