# Supplementary material for: Impact of waiting time for treatment on the progression of superficial esophageal squamous cell carcinoma: a single-center retrospective study
Source: Esophagus. 2025 Sep 17;23(1):174–80. doi: 10.1007/s10388-025-01156-1 (PMC12832587; doi:10.1007/s10388-025-01156-1)
Supplement: Supplementary file 1 — Supplementary file1 (DOCX 21 KB) [file 10388_2025_1156_MOESM1_ESM.docx]

**Supplemental Data 1. Characteristics by Waiting Period in B1-Classified SESCC Cases**

|  | < 1 month | 1-2 months | 2–3 months | >3 months | p |
| --- | --- | --- | --- | --- | --- |
| Age, median (years) | 69 (42–89) | 71 (48–89) | 72 (40–87) | 69.5 (50–79) | 0.51 |
| Sex (M/F), n | 69/9 | 153/14 | 55/4 | 13/1 | 0.77 |
| Location of lesions, n  　O/Ce/Ut/Mt/Lt/Jz  Ant/Right/Post/Left/Circ | 1/3/23/47/23/0  20/21/30/25/1 | 1/4/38/105/51/1  47/44/60/47/2 | 1/6/12/45/8/0  18/16/20/16/2 | 0/1/3/8/4/0  3/5/2/6/0 | 0.40  0.92 |
| Circumferential extent of the lesions, n  　0–1/4, 1/4–1/2, 1/2–3/4, 3/4–1, 1 | 44/45/7/0/1 | 99/78/14/7/2 | 33/30/6/1/2 | 8/3/5/0/0 | 0.07 |
| Tumor size (mm), median | 18 (4–80) | 18 (3–85) | 16 (3–66) | 17 (4–51) | 0.65 |
| Macroscopic type, n  　0–Ⅰ/0–Ⅱa/0–Ⅱb/0–Ⅱc | 1/7/46/43 | 0/12/92/96 | 0/3/37/32 | 0/1/6/9 | 0.85 |
| Conscious sedation/general anesthesia, n | 41/56 | 139/61 | 40/32 | 8/8 | 0.00 |

*O* esophageal orifice, *Ce* cervical esophagus, *Ut* upper thoracic esophagus, *Mt* middle thoracic esophagus, *Lt* lower thoracic esophagus, *Jz* zone of the esophagogastric junction, *Ant* anterior, *Post* posterior, *Circ* circumference,

**Supplemental Data 2. Characteristics by Waiting Period in B2-Classified SESCC Cases**

|  | < 1 month | 1–2 months | 2–3 months | >3 months | p |
| --- | --- | --- | --- | --- | --- |
| Age, median (years) | 66.5 (48–88) | 70.5 (51–89) | 73 (65-89) | 77 (66-86) | 0.07 |
| Sex (M/F), n | 36/2 | 50/4 | 12/2 | 3/1 | 0.44 |
| Location of lesions, n  　O/Ce/Ut/Mt/Lt/Jz  Ant/Right/Post/Left/Circ | 0/0/10/27/6/0  5/7/16/14/1 | 2/2/9/33/9/0  6/13/13/20/3 | 0/0/6/5/3/0  3/4/4/3/0 | 0/0/1/2/1/0  1/1/0/2/0 | 0.62  0.81 |
| Circumferential extent of the lesions, n  　0–1/4, 1/4–1/2, 1/2–3/4, 3/4–1, 1 | 9/23/9/1/1 | 8/26/11/7/3 | 6/4/3/1/0 | 0/4/0/0/0 | 0.25 |
| Tumor size (mm), median | 27 (6–70) | 28 (6–80) | 23 (4–80) | 19 (17–40) | 0.50 |
| Macroscopic type, n  　0–Ⅰ/0–Ⅱa/0–Ⅱb/0–Ⅱc | 0/9/8/26 | 0/6/14/35 | 0/4/5/5 | 0/1/2/1 | 0.28 |
| Conscious sedation/general anesthesia, n | 18/25 | 34/21 | 8/6 | 3/1 | 0.12 |

*O* esophageal orifice, *Ce* cervical esophagus, *Ut* upper thoracic esophagus, *Mt* middle thoracic esophagus, *Lt* lower thoracic esophagus, *Jz* zone of the esophagogastric junction, *Ant* anterior, *Post* posterior, *Circ* circumference,

**Supplemental Data 3. Diagnostic Accuracy of Preoperative IPCL Classification Compared with Histopathological Findings**

Magnifying　endoscopic diagnosis preoperative

Histological diagnosis at the time of ESD

|  | pEP/LPM | pMM/SM1 | pSM2 |
| --- | --- | --- | --- |
| cEP/LPM (B1 vessels) | 96.6% | 3.4% | 0% |
| cMM/SM1 (B2 vessels) | 31.0% | 58.6% | 10.3% |

*EP* carcinoma *in situ*, *LPM* lamina propria mucosa, *MM* muscularis mucosa, *SM1* submucosa to a depth of ≤200 μm from the muscularis mucosa, *SM2* submucosa to a depth >200 μm
